# The Largest Infant on Record

**Published:** 1879-04

**Authors:** 


THE LARGEST INFANT ON RECORD.—Mrs. Captain N. V. Bates, known as Annie Swan, the Nova Scotia giantess, was lately delivered of a child which weighed 23¾ pounds; height, 30 inches; breast measure, 24 inches; head, 19 inches. The child and mother are both doing well. Mrs. Bates' height is seven feet, nine inches, while the little captain measures only seven feet, seven inches in stature.—*News and Miscellany.*

